# Comparison of Gas–Particle Partitioning of Glyoxal and Methylglyoxal in the Summertime Atmosphere at the Foot and Top of Mount Hua

**DOI:** 10.3390/molecules28135276

**Published:** 2023-07-07

**Authors:** Weining Qi, Yifan Zhang, Minxia Shen, Lu Li, Wenting Dai, Yukun Chen, Yali Liu, Xiao Guo, Yue Cao, Xin Wang, Yingkun Jiang, Jianjun Li

**Affiliations:** 1 State Key Laboratory of Loess and Quaternary Geology, Institute of Earth Environment, Chinese Academy of Sciences, Xi’an 710061, China; qweining@126.com (W.Q.); shenmx@ieecas.cn (M.S.); lilu@ieecas.cn (L.L.); daiwt@ieecas.cn (W.D.); chykdhj1993@stu.xjtu.edu.cn (Y.C.); m159299144@163.com (X.G.); lanlantuxinxin@163.com (X.W.); 2 College of Resources and Environment, University of Chinese Academy of Sciences, Beijing 101408, China; 3 Xi’an Institute for Innovative Earth Environment Research, Xi’an 710061, China; yiifanzhang@163.com (Y.Z.); gzslyli@163.com (Y.L.); caoyue16@mails.ucas.ac.cn (Y.C.); yingkunjiang@163.com (Y.J.); 4 State Key Laboratory of Multiphase Flow in Power Engineering, Xi’an Jiaotong University, Xi’an 710049, China; 5 National Observation and Research Station of Regional Ecological Environment Change and Comprehensive Management in the Guanzhong Plain, Xi’an 710499, China

**Keywords:** Gly and mGly, gas-particle partitioning, influencing factors

## Abstract

Glyoxal and methylglyoxal are important volatile organic compounds in the atmosphere. The gas–particle partitioning of these carbonyl compounds makes significant contributions to O_3_ formation. In this study, both the gas- and particle-phase glyoxal and methylglyoxal concentrations at the foot and top of Mount Hua were determined simultaneously. The results showed that the gaseous-phase glyoxal and methylglyoxal concentrations at the top were higher than those at the foot of the mountain. However, the concentrations for the particle phase showed the opposite trend. The average theoretical values of the gas–particle partitioning coefficients of the glyoxal and methylglyoxal concentrations (4.57 × 10^−10^ and 9.63 × 10^−10^ m^3^ μg^−1^, respectively) were lower than the observed values (3.79 × 10^−3^ and 6.79 × 10^−3^ m^3^ μg^−1^, respectively). The effective Henry’s law constants (eff.K_H_) of the glyoxal and methylglyoxal were in the order of 10^8^ to 10^9^ mol/kgH2O/atm, and they were lower at the foot than they were at the top. The particle/gas ratios (P/G ratios) of the glyoxal and methylglyoxal were 0.039 and 0.055, respectively, indicating more glyoxal and methylglyoxal existed in the gas phase. The factors influencing the partitioning coefficients of the glyoxal and methylglyoxal were positively correlated with the relative humidity (RH) and negatively correlated with the PM_2.5_ value. Moreover, the partitioning coefficient of the glyoxal and methylglyoxal was more significant at the top than at the foot of Mount Hua.

## 1. Introduction

Aldehydes and ketones have a carbonyl structure, and so they are called carbonyl compounds (an important class of volatile organic compounds (VOCs) containing oxygen). Common carbonyl compounds mainly include formaldehyde, acetaldehyde, acetone, and so on. Ubiquitous in the atmosphere, they form a vital part of secondary organic aerosols (SOAs) [1,2]. In terms of chemical components, SOAs include organic and inorganic components. Based on the differences in the functional groups they contain, organic matter can be divided into hydrocarbon substances containing C, –OH, –OOH, –CHO, –COOH, etc., while inorganic substances refer to salt substances such as SO_4_^2−^, NO_3_^−^, NH_4_^+^, K^+^, Ca^2+^, Na^+^, Cl^−^, and so on. Carbonyl compounds are mainly derived from the incomplete combustion of fossil fuels and biomass, including automobile exhaust and chemical industry waste gas. Moreover, a photochemical reaction is a vital pathway in the actual atmosphere, and it is not only the significant intermediate product of a hydrocarbon photochemical reaction but also the precursor of generating free radicals, ozone and peroxynitro compounds [3]. However, low molecular-weight carbonyl compounds, such as formaldehyde, are not considered to contribute to SOAs to any significant extent for a long period of time due to their relatively high vapor pressure and, therefore, they have not received much attention [4]. However, Knote et al. have proven that these low molecular-weight carbonyl compounds are highly reactive, and their contributions to SOAs may exceed our recognition [5]. Carbonyl compounds exhibit volatility, and they can be released in the gas phase and enter the particle phase, significantly changing the physico-chemical properties of aerosol particles, such as by reducing the surface tension of aerosol particles to form light-absorbing compounds [6].

Glyoxal (Gly) and methylglyoxal (mGly), the two lower-molecular-weight dicarbonyl compounds, are special substances due to their physico-chemical properties in α-carbonyl compounds. For example, the boiling point of Gly is only 51 °C, making it extremely volatile, and it does not contribute to the mass of an aerosol. However, because of the function of its α-carbonyl group, its reactivity is enhanced, and it reacts readily with other substances. It has been reported that Gly and mGly can react with nitrogen-containing nucleophilic reagents, including ammonia and amine, to produce imine intermediates. In spite of this, the intermediate does not absorb light, and it will continue to react to generate nitrogen-containing organic chromophores [7]. The absorption of brown carbon (BrC) affects the direct radiation effect of an organic carbon in the visible range of solar radiation [8]. The former study indicated that the bleaching of Gly–ammonium sulfate BrC was predominantly driven by OH oxidation, while that of mGly–ammonium sulfate was driven by direct photolysis [9]. Moreover, the high water solubility of low-molecular-weight carbonyl compounds makes them more easily adsorbed on aerosol particles or tiny droplets for liquid-phase chemical reactions [10,11]. Aromatics and biological hydrocarbons with acid-catalyzed heterogeneous reactions produce Gly and mGly [12]. Thus, Gly and mGly are easily distributed into the liquid phase, which contributes to the formation and evolution of SOAs through photolysis, hydrolysis, or polymerization [13,14]. Hu et al. reported that Gly and mGly contributed 14–25% and 23–28%, respectively, to the generation of SOAs in China [15]. The SOA yields of Gly and mGly are significantly dependent on atmospheric conditions, such as salt and water concentrations, and the properties of the surrounding aerosols [16,17]. The sources and fates of Gly and mGly are complex and diverse, and they are affected by multiple environmental factors that cause some uncertainties in the generation of SOAs in the atmosphere.

The gas–particle partitioning of volatile substances can be used to evaluate the contribution of Gly and mGly to SOAs. Water-soluble carbonyl compounds, transforming from the gas to particle phase, follow Pankow’s partitioning theory and Henry’s law. Previous studies have primarily focused on urban areas (Beijing [1,18], Zhengzhou [2], and Denver [19]), and they analyzed the source, distribution, and sink. The findings of the studies are shown in Table 1 and Table 2. Table 1 shows that the gas phase concentrations of Gly and mGly were the highest in summer and the lowest in winter. However, the particle phase, in contrast to the gas phase, had the highest concentrations in winter and the lowest concentrations in summer. The gas–particle partitioning and eff.K_H_ of Gly and mGly for different seasons are shown in Table 2. The gas–particle partitioning of Gly and mGly is related to ambient temperatures, and an increase in temperature is conducive to the volatilization of Gly and mGly from the gas phase to particle phase. Both of these indicate seasonal variations. The main concern is the interconversion of VOCs in the gas phase or PM_2.5_, and their further oxidation to form organic acids with low volatility, such as pyruvate, glyoxylic acid (ωC_2_), and oxalic acid (C_2_) [20], or the two-phase distribution of VOCs in a gas particle state [21]. Few studies [22] on the gas–particle partitioning of carbonyl compounds have been conducted in alpine regions, although these could be helpful in revealing the distribution and transformation mechanisms of these compounds during long-distance transport. Therefore, we conducted simultaneous observations of VOCs and aerosols at both the foot and top of Mount Hua, an alpine region in the Guanzhong Basin, Northwest China. The chemical compositions of the aerosols were analyzed (and have been reported elsewhere [23]), although some of the original data for this article have already been published in Shen et al. [24]. The major purpose of Shen et al. (2023) was to discuss the influence of vertical transport to the formation mechanism of diacids-related SOA from the ground area to the top of Mt. Hua. In this study, in order to explore the gas-particle partitioning of glyoxal and methylglyoxal, as well as its influencing factors, glyoxal and methylglyoxal concentrations in the particle phase were reanalyzed with their concentrations in the gas phase.

## 2. Results and Discussion

### 2.1. Temporal Variations in Gly and mGly Concentrations

**Figure 1:**
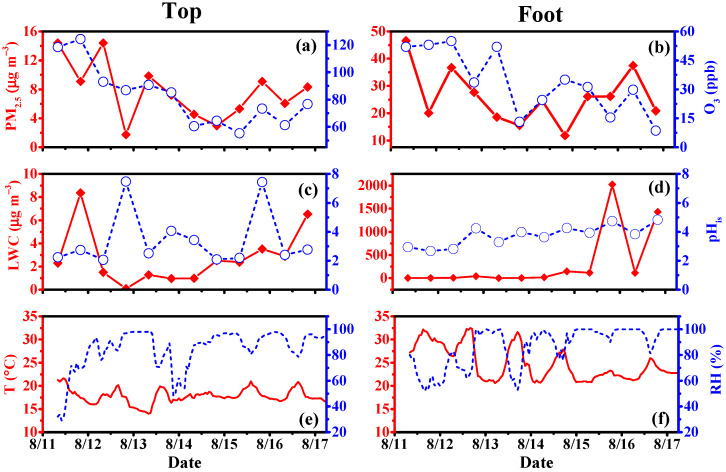
Temporal variations in the concentrations of O_3_, PM_2.5_, liquid water content (LWC), pH_is_, temperature (T), and relative humidity (RH) between the top and foot of Mount Hua in 2020. (**a**,**b**) are the temporal variations in the concentrations of O_3_ and PM_2.5_ between the top and foot of Mount Hua, respectively. (**c**,**d**) are the temporal variations in the concentrations of LWC and pH_is_ between the top and foot of Mount Hua, respectively. (**e**,**f**) are the temporal variations in the concentrations of T and RH between the top and foot of Mount Hua, respectively.

Gly and mGly are the most basic substances in dicarbonyl compounds, which are extremely volatile due to their high vapor pressure. The monophasic (gas phase and particle phase) temporal variations in Gly and mGly are discussed below. The purpose of this section is to elucidate the temporal variation process in the different phase states of Gly and mGly at the top and the foot of Mount Hua. Figure 1 shows the temporal variations in O_3_, PM_2.5_, liquid water content (LWC), in situ acidity (pH_is_), temperature (T), and RH at both the top and the foot of Mount Hua. The concentration of PM_2.5_ at the foot (26.01 ± 10.03 µg m^−3^) was more than three times that at the top (7.75 ± 3.99 µg m^−3^) of the mountain, consisting of the intensive anthropogenic emissions from industries, vehicles, and other human activities at ground level. The concentration of O_3_ at the top (82.49 ± 22.11) was significantly higher than that at the foot of the mountain (33.59 ± 16.49) (Figure 1a,b). There was little difference in the pH_is_ values between the top and the foot of the mountain (Figure 1c,d). The average temperature at the foot was 8 °C higher than that at the top of the mountain. The RH values (49.51 to 99.92%) were relatively high at both sites during the warmer seasons (Figure 1e,f).

#### 2.1.1. Temporal Variations in Gas-Phase Gly and mGly at the Top and Foot of Mount Hua

**Figure 2:**
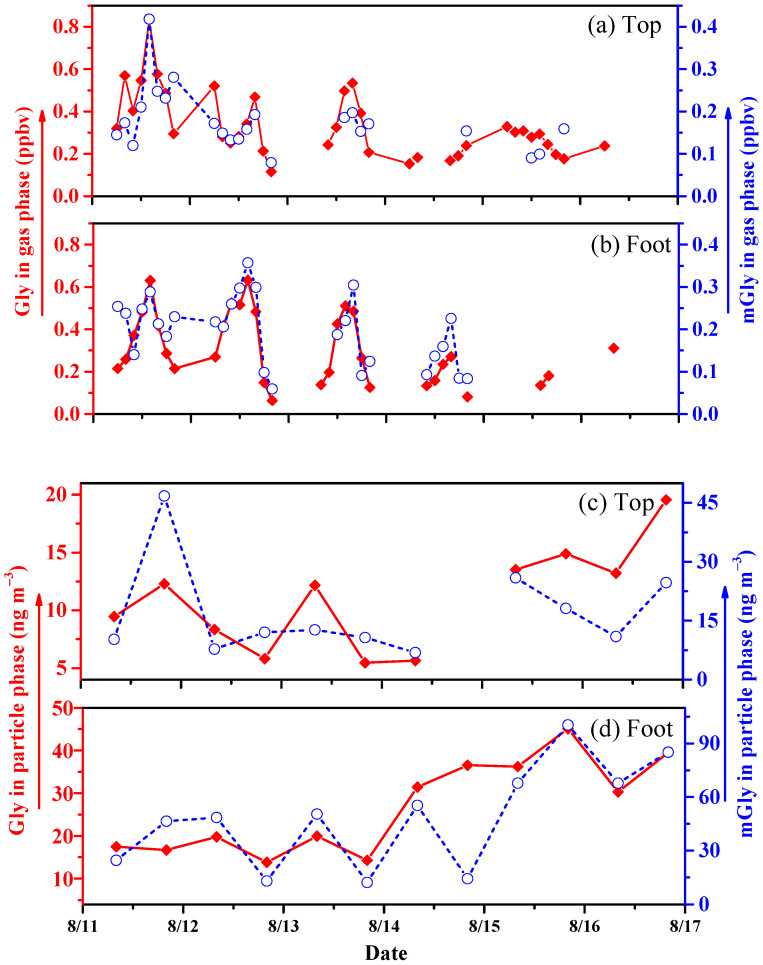
Temporal variations in gas-phase (**a**,**b**) and particle-phase (**c**,**d**) Gly and mGly at Mount Hua in 2020.

Gly and mGly are important carbonyl compounds in the atmosphere and play important roles in free radical cycling and ozone formation [25]. The temporal variations in gas-phase Gly and mGly between the top and foot of Mount Hua are shown in Figure 2a,b. The average concentrations of gas-phase Gly and mGly were 0.28 ± 0.13 and 0.17 ± 0.06 ppbv, respectively, at the top and 0.23 ± 0.14 and 0.16 ± 0.07 ppbv, respectively, at the foot of the mountain. The difference in total concentration was negligible, but the processes of the change were markedly different. Gly and mGly showed similar trends at both sites. The concentrations of gas-phase Gly and mGly both increased gradually in the afternoons (12:00–16:00) and decreased in the evenings. The concentrations of Gly were higher than those of mGly, which may have been related to the shorter lifetime of mGly compared to that of Gly (mGly 1.6 h vs. Gly 2.9 h) [22]. Furthermore, the concentration of Gly at the top was higher than that at the foot of the mountain during the first day of sampling. For this reason, there was thought to be a process of the mutual transformation of mGly to Gly with the effect of the high O_3_ concentrations at the top of the mountain. Furthermore, the top of the mountain, with its high levels of plant-source VOCs and low NO*_x_*, was conducive to the formation of O_3_ [26]. Gly reached its maximum gas-phase value on day 11 of the sampling period (mainly because of the conditions of strong solar radiative forcing during sunny days, which were more conducive to the gas-phase reactions of Gly than those on rainy days (i.e., the subsequent sampling days)).

#### 2.1.2. Temporal Variations in Particle-Phase Gly and mGly at the Top and Foot of Mount Hua

The temporal variations in particle-phase Gly and mGly at the top and foot of Mount Hua are shown in Figure 2c,d. The particle-phase amounts of Gly and mGly at the foot were 26.75 ± 10.98 and 48.80 ± 28.85 ng m^−3^, respectively, which were two to three times higher than those at the mountaintop (10.94 ± 4.45 and 16.97 ± 11.73 ng m^−3^, respectively). The LWC at the foot of the mountain was also higher than that at the top. It has been reported that carbonyl compounds will occur in aqueous-phase reactions when dissolved in liquid water and concentrated salt solutions [3]. VOCs can be converted into high-oxygen and low-volatile organic acids during aqueous-phase chemical processes, which contributes to increases in the contents of SOAs in the environment with the evaporation of water [27]. Gly and mGly will generate hydroxyhydroperoxide intermediates during the aqueous-phase process, which will reversibly generate Gly and mGly again after H_2_O_2_ quenching [3]. As a result, with the increase in LWC, mGly and Gly both increase significantly. The growth trend at the foot of the mountain was greater than that at the top. Compared with the top of mountain, Gly and mGly had more anthropogenic sources at the foot of the mountain, such as vehicle exhaust emissions, temple incense-burning activities, restaurant cooking, and firecrackers.

Different from gas-phase Gly, the particle-phase Gly amounts were significantly lower than those of mGly during the first two days and, later, the contents of Gly were higher than those of mGly, showing an upward trend. The concentrations of mGly at the foot of the mountain were higher than those of Gly. The reason for this may have been that the methylation of Gly molecules occurs readily in aqueous-phase chemical reactions, forming mGly. These results were comparable with those of Zhu et al. recorded at Mount Tai in 2014 [28], and they were higher than those found by Meng et al. in 2016 [29].

### 2.2. Gas–Particle Partitioning of Gly and mGly at the Top and Foot of Mount Hua

#### 2.2.1. Measured and Theoretical Partitioning Coefficients

Carbonyl compounds are ubiquitous in the atmosphere and are important components of atmospheric aerosols; however, their actual contribution to SOAs is difficult to quantify because they exist in both the gas and particle phases, coupled with the highly volatile nature of the substances themselves. Pankow’s absorptive partitioning theory is used to describe the Gly and mGly gas–particle partitioning process to reveal the mechanism of the generation of SOAs. The average theoretical values of Gly and mGly are 4.57 × 10^−10^ m^3^ μg^−1^ and 9.63 × 10^−10^ m^3^ μg^−1^, respectively, while the actual in situ measurements were 3.79 × 10^−3^ m^3^ μg^−1^ and 6.79 × 10^−3^ m^3^ μg^−1^, respectively (Table 3). The observed values were six orders of magnitude higher than the theoretical values. Furthermore, the partitioning coefficients of the field observations at the top of the mountain were higher than those at the foot of the mountain. Meanwhile, the temperatures at the foot of the mountain were 8 °C higher than those at the top of the mountain. High temperatures promote the volatilization of carbonyl compounds into the gas phase, resulting in decreases in the adsorption coefficients [30]. Therefore, temperature affected the distribution coefficients. The partitioning coefficient of mGly at the top was one order of magnitude higher than that at the foot of the mountain, and it was greater than that of Gly, suggesting that mGly, in mountain regions, is more prone to phase transfer than Gly.

It has been reported that the $K_{p}^{f}$ values of Gly and mGly were 1.44 × 10^−3^ and 4.19 × 10^−4^ m^3^ μg^−1^, respectively, in the Beijing urban area [1], and they were 4.44 to 7.34 × 10^−5^ and 0.68 to 1.14 × 10^−5^ m^3^ μg^−1^, respectively, in the photochemical reaction products of isoprene in the laboratory [31]. The relatively higher $K_{p}^{f}$ values in this study may have been related to the differences in the chemical compositions and sources of the aerosols. Consistently, these results indicated that Pankow’s absorptive partitioning theory has been markedly underestimated in field and laboratory studies, meaning that Pankow’s absorptive partitioning theory cannot predict the gas–particle partitioning of Gly and mGly [1,32].

#### 2.2.2. Gas–Particle Partitioning Ratios and Henry’s Law

Table 4 shows that the P/G ratios of Gly and mGly at night are higher than those during the day at the top of Mount Hua in the summer, and this change was similar to the change in RH. Our results were consistent with previously reported results from the summit of Mount Fuji [22]. On the contrary, the P/G ratios of mGly during the day were higher than those at night at the foot of the mountain. The average P/G ratios at the foot of the mountain were higher than those at the top of the mountain due to the vertical downward transport of the particle phase. Furthermore, the P/G ratios were generally small (0.016 to 0.0961), which indicated that these two carbonyl compounds were volatile, and most of them were present in the gas phase. Therefore, gas–particle partitioning should be considered when using particle concentrations in source apportionments [33,34]. The eff.K_H_ values of Gly and mGly at night (2.88 × 10^9^ and 6.82 × 10^9^, respectively) were higher than those during the day (4.45 × 10^8^ and 9.81 × 10^8^, respectively), and the values at the top were higher than those at the foot of Mount Hua. Moreover, the eff.K_H_ values of Gly and mGly at the top (1.66 × 10^9^ and 4.22 × 10^9^, respectively) were an order of magnitude higher than those at the foot (2.51 × 10^8^ and 6.39 × 10^8^, respectively) of the mountain. Our results were of the same order of 10^8^ to 10^9^ as those previously reported [2]. This value was much higher than the theoretical value primarily because the particles were formed based on a concentrated salt solution [35], and their formation conditions were distinct from those of an infinite dilute solution. Moreover, carbonyl compounds interact with other aerosols, such as oxygenated organic aerosols [36] and aerosol liquid water, which were not excluded in the estimation.

### 2.3. The Factors Influencing the Gas–Particle Partitioning Process

It has been reported that the gas–particle partitioning of carbonyl compounds is affected by RH [38,39], particle acidity [40], and inorganic ions [41,42], especially during the formation of SOAs. This was key to understanding the formation of SOAs to explore the factors influencing the gas–particle partitioning of carbonyl compounds at Mount Hua, and so a comparative analysis of the following systems was conducted.

**Figure 3:**
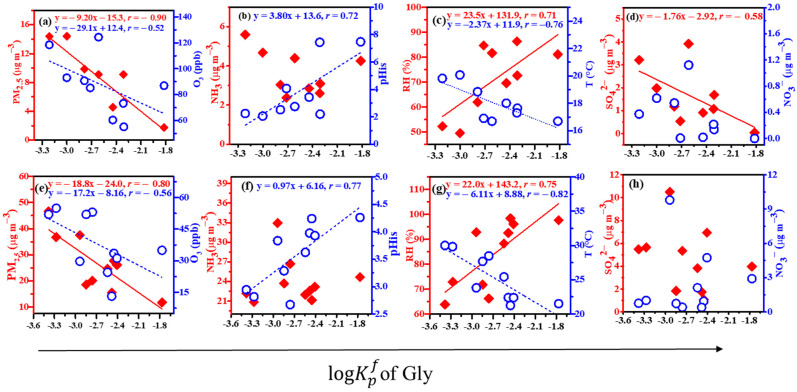
Linear correlations between the $\log K_{p}^{f}$ values of Gly and the PM_2.5_, O_3_, NH_3_, pH_is_, RH, T, SO_4_^2−^, and NO_3_^−^ at the top (**a**–**d**) and the foot (**e**–**h**) of Mount Hua (for all of the above data, *p* < 0.01).

**Figure 4:**
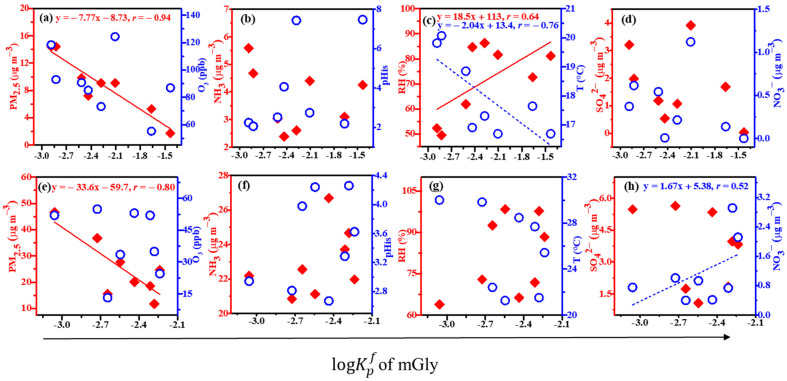
Linear correlations between the $\log K_{p}^{f}$ values of mGly and the PM_2.5_, O_3_, NH_3_, pH_is_, RH, T, SO_4_^2−^, and NO_3_^−^ at the top (**a**–**d**) and the foot (**e**–**h**) of Mount Hua (for all of the above data, *p* < 0.01).

Figure 3 and Figure 4 show the linear correlations between the $\log K_{p}^{f}$ values of Gly and mGly and the PM_2.5_, O_3_, NH_3_, pH_is_, RH, T, SO_4_^2−^, and NO_3_^−^ at the top (a, b, c, and d) and the foot (e, f, g, and h) of Mount Hua. The results indicated that the $\log K_{p}^{f}$ value of Gly was negatively correlated with the PM_2.5_, O_3_, and T, which were positively correlated with the pH_is_ and RH at the top and foot of the mountain and negatively correlated with the SO_4_^2−^ at the top of Mount Hua. The $\log K_{p}^{f}$ values of mGly at the top and foot of Mount Hua were significantly negatively correlated with the PM_2.5_, and the linear correlation coefficients (*r*) were −0.94 and −0.80, respectively. In addition, the peak also had a strong correlation with the *T* (negative correlation) and RH (positive correlation) while the foot was positively correlated with the NO_3_^−^. It has been reported that temperature is negatively correlated with the partition coefficients of carbonyl compounds [2]. Our results were consistent with those of the previous study.

The higher the concentrations of the PM_2.5_ and O_3_, and the higher the temperature *T*, the more adverse the effects on the process of the gas–particle partitioning of Gly within the scope of this observation. The average PM_2.5_ values at the top and bottom of the mountain were 26.01 ± 10.03 µg m^−3^ and 7.75 ± 3.99 µg m^−3^, respectively. The correlations between the PM_2.5_ and Gly (*r* = −0.90) and mGly (*r* = −0.94) at the top were closer to 1 than those at the foot (*r* = −0.80 and −0.80, respectively). The higher the PM_2.5_, the more the Gly and mGly molecules coated the surfaces of the particulate matter, hindering the gas–particle partitioning process. Hence, the partitioning effects on Gly and mGly at the top of the mountain were more favorable than those at the bottom. The linear correlation analysis showed that high O_3_ would inhibit the gas–particle partitioning of Gly. As atmospheric oxidation increased, Gly could be further oxidized to form fewer volatile organic acids, such as C_2_ [43].

It has been reported that RH and the particle-phase state may play key roles in the heterogeneous oxidation of organic–inorganic mixed aerosols [44]. Our observations confirmed this finding. In the particle phase, when RH is high, Gly and mGly are adsorbed on particles and react with water to form hydrates, and they can further react with the hydroxyl group of glycols to produce dimers, trimers, and even polymers with higher molecular weights. The hygroscopicity of aerosols increases exponentially with increases in RH [35]. As a result, the hygroscopic growth of aerosols and the hydration of Gly and mGly can accelerate their distribution in the particle phase at relatively high RH levels. However, the hydrates of Gly and mGly can form even in the gas phase, with hydrated Gly and mGly having greater affinities for water vapor and water droplets than anhydrous compounds [45]. Whether in the gas phase or the particle phase, an increase in RH is conducive to the formation of Gly and mGly. The partitioning coefficients at the top and bottom of the mountain for the $\log K_{p}^{f}$ values of Gly had good correlations with the acidity of the aerosol particles. The same experimental results have been reported elsewhere [46]. Inorganic substances always coexist with organic substances in atmospheric particulates and, together, they affect the heterogeneous oxidation of organic aerosols [47]. The water content and phase state of particles may change significantly in the presence of inorganic salts [48,49,50]. Jang et al. observed, in a laboratory, that the O_3_ reaction of isoprene and acrolein produces Gly, and that H_2_SO_4_ can catalyze the heterogeneous carbonyl reaction, leading to increases in SOAs [12]. The linear correlation coefficient of the $\log K_{p}^{f}$ value of Gly and SO_4_^2−^ was −0.58 at the top of the mountain, and that of the $\log K_{p}^{f}$ value of mGly and NO_3_^−^ was 0.52 at the bottom of the mountain in this observation. This suggested that SO_4_^2−^ and NO_3_^−^ have certain effects on the gas–particle partitioning of Gly and mGly.

## 3. Materials and Methods

### 3.1. Field Sampling

The sampling campaign was conducted at the top (*c*. 2060 m a.s.l) and at the foot (*c*. 400 m a.s.l) of Mount Hua (34°29′ N, 110°05′ E) from 11 to 16 August 2020. The gas-phase samples were collected using a Sep-Pak DNPH-Silica Plus Short Cartridge, 350 mg Sorbent per Cartridge, 55–105 µm, 20/pk (Waters 037500) with a Sep-Pak Ozone Scrubber Potassium Iodide, Plus Short Cartridge, 1.4 g, 55–105 µm, 20/pk (Waters 054420). Potassium iodide (KI) was connected to its front to eliminate ozone interference. The flow rate was 0.6 L min^−1^. The sampling duration was 2 h between 06:00 and 20:00 and 10 h between 20:00 and 06:00. The PM_2.5_ samples were collected using a mid-volume air sampler (HC-1010, Qingdao Hecheng Environmental Protection Technology Co., Ltd., Qingdao, China) equipped with prebaked (450 °C, 8 h) quartz-fiber filters (Whatman, 90 mm diameter, Clifton, NJ, USA) at an airflow rate of 100 L min^−1^. The sampling time was 11 h by day (08:00–19:00) and by night (20:00–07:00). The meteorological parameters (relative humidity (RH), temperature [51], etc.) at the top and foot of Mount Hua were taken from local meteorological department data.

### 3.2. Sample Extraction and Analysis

The gaseous carbonyl compounds were collected using a 2,4-dinitrophenylzine (DNPH) (Sep-Pak DNPH-silica, 55–105 μm particle size, 125 Å pore size; Waters Corporation, Milford, MA, USA) adsorption column. The collected samples were slowly washed with 2 mL acetonitrile to ensure that all samples were washed to the volumetric bottle. Then, high-performance liquid chromatography (HPLC) with ultraviolet detection (Agilent 1200 LC; Agilent Technology, Santa Clara, CA, USA) was used for the subsequent assay. The analytical conditions involved a PerkinElmer Spheri-5 ODS reversed-phase column (250 mm × 4.6 mm, 5.0 μm, PerkinElmer, Norwalk, CT, USA) and the mobile phases were as follows: mobile A: water/CH_3_CN/tetrahydrofuran (60/30/10 *v/v*), mobile B: CH_3_CN/water (60/40, *v/v*), and mobile C: CH_3_CN (100 v). The detection wavelength was 360 nm, the column temperature was 25 °C, the injection volume was 20 mL, and the flow rate was 2 mL min^−1^. The gradient elution procedure was: 80% mobile A/20% mobile B for 1.5 min; 40% mobile A/60% mobile B for 5 min; 100% mobile B for 8.5 min; 80% mobile B/20% mobile C for 3 min; 50% mobile B/50% mobile C for 4 min; and 100% mobile C for 6 min.

The extraction method for particle phase was adopted from the study by Shen et al. [24]. In short, a one-quarter filter membrane was used for the ultrasonic extraction (three cycles of 15 min each) with ultra-pure water. The water extract was cyclically steamed until it was near-dry, then it was treated with 14% BF_3_/n-butanol at 100 °C for 1 h and extracted. Finally, the extract was concentrated to 100 μL and analyzed by gas chromatography (GC; HP 6890, Agilent Technology, Santa Clara, CA, USA). The method detection limit (MDL) was 0.1 ng m^−3^. In this study, the concentration at 3 times the signal-to-noise ratio was determined as the MDL, and the analysis error of the repeated analysis was 15%.

The methods of determination of the O_3_, water-soluble inorganic ions, and other chemical compounds, and the calculations for the LWC and pH_is_ in the PM_2.5_, can be found in our previous study [24].

### 3.3. Estimation of the Partitioning Coefficients

In this study, the partitioning coefficients of the compounds were calculated using Pankow’s absorptive partitioning theory (Equations (1) and (2)) and Henry’s law (Equation (3)). They are expressed as follows:

(1) $$K_{p}^{f}=\frac{C_{p}}{C_{g}\times \;\mathrm{TSP}}$$

(2) $$K_{p}^{t}=\frac{RTf_{\mathrm{om}}}{10^{6}MW_{OM}\zeta P_{L}^{0}},\;\mathrm{and}$$

(3) $$\mathrm{eff}.K_{H}=\frac{C_{\mathrm{aw}}}{P}=\frac{C_{P}}{\mathrm{LWC}}\frac{P_{T}}{C_{g}\mathrm{RT}}$$

In Equation (1), $K_{p}^{f}$ (m^3^ µg^−1^) denotes the field-measured gas–particle partitioning coefficient, *C_p_* (µg m^−3^) refers to the concentration of dicarbonyls in the particle phase, *C_g_* (µg m^−3^) is the concentration of the dicarbonyls in the gas phase, and TSP (µg m^−3^) is the mass concentration of the suspended particles (the mass concentrations of the PM_2.5_ were used in this study). In Equation (2), $K_{p}^{t}$ (m^3^ µg^−1^) is the theoretical gas–particle partitioning coefficient determined by Pankow’s absorptive model, *f*_om_ is the absorbing fraction of the total particulate mass, MW_OM_ (g mol^−1^) denotes the mean molecular weight of the organic phase, and *ζ* is the activity coefficient of the target compounds. In the estimation of $K_{p}^{t}$ in this study, *f*_om_ and *ζ* were unity and MW_OM_ was 200 g mol^−1^, as has been used in previous studies [18,52]. The vapor pressure ($P_{L}^{0},$ the primary determinant in the estimation of $K_{p}^{t}$) can be calculated using the extended aerosol inorganic model (E-AIM). In Equation (3) [22], *C_aw_* is the concentration of the aerosol water (molkg^−1^ H_2_O), *P* is the partial pressure of the compound (atm), *C_p_* is the particulate carbonyl concentration (ng m^−3^), LWC is the aerosol liquid water (g m^−3^), *P_T_* is the total atmospheric pressure (atm), *C_g_* is the gaseous carbonyl concentration (ng m^−3^), *R* represents the ideal gas constant (0.0821 L atm (K mol) ^−1^), and *T* is the ambient temperature (K).

### 3.4. Quality Assurance and Quality Control

Since carbonyl compounds are highly volatile and widely present in the atmospheric environment, to reduce experimental errors, the following treatments were conducted before and after the experiment. Before the experiment, flow calibrations and air tightness tests were conducted on the sampler for collecting the gas phase and particle phase samples, and the quartz-filter membrane was burned at a high temperature (450 °C for 8 h). After sampling, the gas-phase sampling head and ozone column were sealed and refrigerated at 4 °C, and the particle-phase samples were sealed with aluminum foil and refrigerated at −20 °C. Blank gas-phase samples were collected by placing a blank DNPH cylinder near the gas inlet for the same time without manual suction. The blank particle-phase sample was collected by placing a blank quartz filter at the PM_2.5_ inlet and collecting the blank sample in the field for approximately 10 min without sucking air. All data used in this study were corrected with reference to the blank sample.

## 4. Conclusions

The gas- and particle-phase concentrations, the gas–particle partitioning process, and the factors influencing the concentrations of Gly and mGly at the top and foot of Mount Hua were analyzed. The gas-phase concentrations of Gly and mGly at the top were higher than those at the foot of Mount Hua while the particle-phase concentrations at the foot were higher than at those at the top of Mount Hua. The field observation values for the Pankow’s absorptive partitioning coefficients and Henry’s law constants were 6 and 4–5 orders of magnitude higher than the theoretical values, respectively, and they did not reflect the distribution of Gly and mGly in the atmosphere. The P/G ratios showed that more Gly and mGly were present in the gas phase, confirming that these low-molecular-weight carbonyl compounds can easily volatilize from the particle phase into the gas phase. It is unreasonable to consider only the concentrations of these compounds in the particle phase to analyze their source distributions in the atmosphere. The factors affecting the gas–particle partitioning of volatile materials are complicated. Our research results indicated that RH and PM_2.5_ affected the two-phase partition transition of Gly and mGly on Mount Hua, and these effects were more significant at the top than at the foot of the mountain. Based on the findings of this study, future research should aim to ascertain the difference between the partitioning coefficients of Gly and mGly and determine the causes of this phenomenon in order to narrow the gap between the theoretical and the observed values in the field.

## Figures and Tables

**Table 1:** Concentrations of Gly and mGly during different seasons.

Season	Gas Phase (ppbv)		Particle Phase (ng m^−3^)		References
	Gly	mGly	Gly	mGly	
Spring	0.02 ± 0.02	0.12 ± 0.08	15.24 ± 17.50	6.07 ± 2.79	[18]
Summer	0.13 ± 0.07	0.87 ± 0.54	10.18 ± 6.63	9.50 ± 5.62	
Autumn	0.07 ± 0.03	0.15 ± 0.09	9.33 ± 4.24	9.15 ± 3.62	
Winter	0.06 ± 0.05	0.11 ± 0.09	28.77 ± 25.33	14.61 ± 10.15	
Autumn	0.10	0.31	12.01	8.27	[1]
Spring	0.17 ± 0.10	0.32 ± 0.14	61.46 ± 16.26	64.33 ± 6.78	[2]
Summer	0.30 ± 0.13	0.90 ± 0.35	21.33 ± 11.81	17.10 ± 9.28	
Autumn	0.18 ± 0.05	0.59 ± 0.15	30.88 ± 16.72	25.42 ± 13.53	
Winter	0.11 ± 0.04	0.37 ± 0.15	61.46 ± 32.30	64.33 ± 33.18	

**Table 2:** $K_{p}^{f}$ and eff.K_H_ of Gly and mGly during different seasons.

Season	$K_{p}^{f}$		eff.K_H_ (M atm^−1^)		References
	Gly	mGly	Gly	mGly	
Spring	1.43 × 10^−2^	1.06 × 10^−3^	2.53 × 10^9^	1.33 × 10^8^	[18]
Summer	8.11 × 10^−4^	1.49 × 10^−4^	1.96 × 10^8^	4.92 × 10^7^	
Autumn	2.14 × 10^−3^	9.55 × 10^−4^	5.08 × 10^8^	8.52 × 10^7^	
Winter	1.30 × 10^−2^	2.60 × 10^−3^	1.04 × 10^9^	2.63 × 10^8^	
Autumn	1.44 × 10^−3^	4.19 × 10^−4^	1.66 × 10^9^	5.16 × 10^8^	[1]
Spring	1.33 × 10^−3^	3.48 × 10^−4^	2.20 × 10^9^	6.88 × 10^8^	[2]
Summer	6.31 × 10^−4^	1.40 × 10^−4^	2.85 × 10^9^	4.81 × 10^8^	
Autumn	1.05 × 10^−3^	2.07 × 10^−4^	2.53 × 10^10^	4.95 × 10^9^	
Winter	1.71 × 10^−3^	4.27 × 10^−4^	1.07 × 10^9^	2.44 × 10^8^	

$\;K_{p}^{f}$: field-measured gas–particle partitioning coefficient.

**Table 3:** Field-derived $K_{p}^{f}$ values for Gly and mGly compared with the theoretically predicted $K_{p}^{t}$ values (m^3^ µg^−1^).

Position	Compound	$K_{p}^{f}$ Average	$K_{p}^{t}$	$K_{p}^{f}$ $/K_{p}^{t}$
Top	Gly	4.02 × 10^−3^	5.32 × 10^−10^	7.55 × 10^6^
	mGly	1.01 × 10^−2^	1.12 × 10^−9^	9.08 × 10^6^
Foot	Gly	3.59 × 10^−3^	3.83 × 10^−10^	9.40 × 10^6^
	mGly	3.43 × 10^−3^	8.08 × 10^−10^	4.24 × 10^6^
Total	Gly	3.79 × 10^−3^	4.57 × 10^−10^	8.30 × 10^6^
	mGly	6.79 × 10^−3^	9.63 × 10^−10^	7.05 × 10^6^

**Table 4:** Partitioning (P/G ratios) for Gly and mGly between the particle and gas phases and the estimated effective Henry’s law constants.

	Top		Foot	
	Gly	mGly	Gly	mGly
P/G ratios				
Daytime	0.016 ± 0.0062	0.0460 ± 0.0460	0.0468 ± 0.0333	0.0857 ± 0.0430
Nighttime	0.0266 ± 0.0128	0.0525 ± 0.0199	0.0961 ± 0.0747	0.0622 ± 0.0194
Average	0.0188 ± 0.0119	0.0439 ± 0.0350	0.0665 ± 0.0559	0.0740 ± 0.0333
N/D ratio	0.799 ± 0.578	1.333 ± 1.519	1.583 ± 0.951	8.022 ± 7.968
Effective Henry’s law constant (mol/kgH_2_O/atm)				
Daytime	4.45 × 10^8^	9.81 × 10^8^	2.21 × 10^8^	9.67 × 10^8^
Nighttime	2.88 × 10^9^	6.82 × 10^9^	2.91 × 10^8^	3.10 × 10^8^

Abbreviations: P/G ratios, particle/gas ratios; N/D ratio, the nighttime/daytime partitioning ratios; P/G ratio at nighttime (8 p.m.−7 a.m.) versus daytime (8 a.m.−7 p.m.). The theoretical Henry’s law coefficients (K_H_) of Gly and mGly were 4.14 × 10^5^ and 3.74 × 10^4^ (295 K), respectively [37].

## Data Availability

Data used in this study are available by request from the corresponding author.
